# The impact of carbon emissions from lag fertilization on wheat production

**DOI:** 10.1371/journal.pone.0299299

**Published:** 2024-03-21

**Authors:** Atif Rahim, Qianrui Peng, Huashuai Chen, Yuxi Liu

**Affiliations:** 1 Business School of Xiangtan University, Xiangtan, P.R China; 2 School of Economics, Hunan University of Finance and Economics, Changsha, P.R China; 3 Suzhou North America High School, Suzhou, P.R China; HNBGU: Hemvati Nandan Bahuguna Garhwal University, INDIA

## Abstract

This study examines the influence of lag fertilization techniques on Pakistani wheat production, highlighting the need to understand and mitigate the environmental impacts of farming methods. The basic purpose of this study is to investigate the impact of CO2 emission from fertilization and other factors on wheat production in Pakistan, using a time series of data from 1990 to 2020. CO2 emission from fertilization (CO2EF) is estimated using the default values provided by the IPCC guidelines. The ARDL approach analyses the short-run and long-run effects of CO2EF, technology level, energy use, agricultural land, and agricultural labor on wheat production. The results show that all factors have significantly impacted wheat production in Pakistan at levels of 1% and 5% significance, both in the short and long run. These findings suggest that reducing CO2EF, technology level, energy use, agricultural land, and agricultural labor on wheat production can help to increase wheat production in Pakistan. The study also highlights the importance of adopting sustainable and efficient fertilization practices, exploring alternative fertilizers, and using crop rotation systems to mitigate the adverse effects of carbon emissions from nitrogen fertilization, energy use, and the use of technology. These measures can contribute to a more sustainable and climate-resilient agriculture sector in Pakistan.

## Introduction

Over the past ten years, there has been a growing concern about how climate change affects the worldwide food supply [1, 2]. The release of excessive greenhouse gases, particularly carbon dioxide, leads to ecological deterioration and alterations in weather patterns. Any disruption in wheat production could have significant consequences for food security and the economies of developing nations, such as Pakistan, which depend heavily on agriculture [3, 4]. Agriculture’s fundamental role in maintaining human life and prosperity has been firmly entrenched in people’s cultural practices and economic systems throughout history [5, 6]. Agriculture accounts for over 21% of Pakistan’s GDP and employs over 70% of the rural population. Despite its significance to the overall economy, the agricultural sector is in decline due to various issues. These issues are exacerbated by urbanization, overpopulation, more excellent fertilizer applications, reliance on traditional agricultural systems, carbon emissions, and climate change [7].

Farmers must transition from conventional to modern agricultural practices to enhance crop yields, meet expanding wheat demand, and maintain natural resources [4, 8]. The relationship between farming practices and carbon emissions is becoming increasingly important in the context of global agriculture and climate change. Pakistan, an agrarian nation heavily dependent on wheat production, faces the challenge of ensuring food security while mitigating the impact of agriculture on the environment [9]. Agricultural activities contribute significantly to the world’s greenhouse gas emissions, with synthetic fertilizers being identified as a significant source. Nitrous oxide (N2O), a potent greenhouse gas, results from the interactions between nitrogen-based fertilizers and the soil’s microbial processes [10].

Traditional fertilizer application methods can cause excess nitrogen in the soil, leading to increased carbon emissions and exacerbating the worldwide conundrum of climate change [11]. Fertilization has emerged as a promising solution to this problem. By precisely applying nutrients at the optimal growth stages of the wheat crop, fertilization improves nutrient utilization, reduces environmental nutrient losses, and reduces carbon emissions [12]. Wheat is a staple food crop in Pakistan, providing sustenance to a significant portion of its population. Therefore, it is essential to enhance wheat production for food security and economic stability. However, there still needs to be a research gap in understanding how fertilization affects carbon emissions and sustainability in Pakistan’s wheat production. A more comprehensive understanding of how lag fertilization, carbon emissions, and wheat production in Pakistan interact is necessary to make sustainable agricultural policies. This research aims to fill this gap by thoroughly investigating the impact of carbon emissions from lag fertilizer, energy consumption, fertilizer application, and technology use (tractors) in Pakistani wheat production. Our study will examine the complex process of lag fertilization, examine the underlying mechanisms controlling carbon emissions, and critically analyze existing literature to identify gaps and limitations. By discussing theories, examining real-life examples, and reviewing what is already known, we aim to provide a complete picture of how lag fertilization, carbon emissions, and wheat production in Pakistan are interconnected. This information can help policymakers, agronomists, and researchers align farming practices with food security and reduce climate change.

### Literature review

By the beginning of the 20^th^ century, agricultural productivity had grown by utilizing more inputs to keep up with the expanding population, resulting in higher carbon emissions from the agricultural industry [13]. Consequently, farming has become the single most significant cause of air pollution, including carbon emissions from livestock, soil deterioration from excessive fertilizer use, and rice production [7]. Several expensive mitigation measures have been offered, indicating the need to alter agricultural systems to reflect evolving demand and resource depletion patterns. It is unclear how agricultural production relates to agricultural carbon emissions [14]. Farmers have always emphasized output expansion above environmental sustainability and soil fertility. Nitrogen emissions increased due to the radiation effects of fertilizer overuse [15]. The growing usage of fossil fuels in agriculture, particularly power electric tractors, has exacerbated the situation. Several researchers examined carbon emissions, energy use, and agricultural productivity [16, 17]. Early studies considered energy utilization, carbon dioxide emissions, and technical efficiency in potato cultivation in Iran. In wheat agriculture, positive and statistically significant connections were discovered between fertilizer, irrigation water, and energy utilization [18]. A study conducted in Thailand looked at the link between carbon dioxide (CO2) emissions, energy use, and wheat production and their impacts on farm economics and climate change. The findings revealed a link between excessive energy use, technological development, and environmental and economic implications [19, 20]. Fossil fuels met most of agriculture’s energy demands. Lowering fertilizer and energy consumption, on the other hand, may boost wheat yield, leading to more concentrated food production and fewer emissions [21]. The researcher Bakhsh [22] assessed the relationship between open farms in Iran’s energy use, GHG emissions, and wheat yield. According to their findings, reducing energy waste significantly reduces greenhouse gas emissions. Apergis & Payne [23], also explored the dynamic relationship between wheat production, energy use, and fertilizer from 1980 to 2013, agriculture employment in Sub-Saharan Africa is examined using panel data. Overall, the data demonstrated that energy use, agriculture employment, fertilizer, and carbon emission negatively and significantly impact wheat production. Several studies have shown that carbon emissions, fertilizer usage, fertilizer intake, crop production, and technical efficiency are positively connected [24, 25]. Researchers have used various models to investigate the linear and non-linear interactions between study variables. For example, Bölük & Mert, [26] investigated the relationship between energy consumption, agricultural economic growth, and the application of the ARDL model using time series data from 1984 to 2016. Their study found that energy utilization favors agricultural economic growth in both the long and short term. According to another researcher [10], in a Pakistani study, changes in energy use (such as gasoline and gas) significantly influence wheat production. Begum [11] recently employed Quantile ARDL to examine the impact of Turkey’s utilization of renewable and non-renewable energy sources on the country’s ecological footprint. Non-renewable energy use was shown to be negatively associated with renewable energy consumption in wheat production. Using the ARDL bound testing model, Khoshnevisan [12] calculated the impact of energy consumption and CO2 emissions on wheat production. Long-term links have been identified between energy use and carbon dioxide emissions in wheat production. According to the study Dogan & Seker [27], panel data assessed agriculture employment, energy use, technology, and carbon emissions impact on wheat production in South Asian countries from 1980 to 2014. The findings revealed a U-shaped trend in reverse, suggesting a relationship between wheat production and carbon emissions. Several authors have utilized the ARDL model to investigate the relationship between CO2 emissions and external variables. In research undertaken by Mohammadi [28], globalization of energy use, fertilizer, and CO2 were all demonstrated to have a positive and statistically significant short-term influence on wheat production. Asongu [29] utilized the NARDL model to investigate the relationship between energy use, carbon dioxide emissions, and wheat production. The study discovered a non-linear and asymmetric link between using different energy sources and their impact on wheat production. Examples of these energy sources are crude oil, electricity, and fossil fuels. Furthermore, according to Xu [30], the NARDL model, non-renewable energy consumption had a significant negative impact on wheat yield, even though the relationship between carbon emissions and economic development was asymmetric, being influenced equally by positive and negative shocks. CO2 emissions in Pakistan were shown to be greater following industrialization and fertilizer use. The NARDL model also demonstrated the short-run effects of energy use and CO2 emissions on South African wheat production, proving the presence of energy consumption and actual output). Numerous studies have approximated one-way or two-way asymmetry of CO2 emissions in response to positive or negative changes in external causes using panel data or time-series data for different countries and historical eras [31]. According to research Wang et al., [32], the energy required and emissions produced throughout the wheat-growing process in Pakistan must be identified. The impact of fertilizers, mechanization, and other factors on agricultural carbon emissions is investigated. This research shows that implementing environmentally responsible agriculture practices may reduce CO2 emissions while increasing wheat productivity. The effects of fertilizer application on soil fertility and wheat productivity were investigated). It investigates the relationship between greenhouse gas emissions and long-term agricultural sustainability. According to the results, fertilizer usage is critical to preserving agricultural productivity and ecological balance. According to Wang et al., [33]), using the Tapio model, the carbon emissions from crop planting in 31 Chinese provinces from 2000 to 2021. It found a fluctuating decline in carbon emissions from 2000 to 2021, weakly decoupling from grain production. However, a strong decoupling was achieved in 2021. A 1% increase in planting scale led to 1.0%, 0.68%, and 0.31% increases in carbon emissions from plotting, irrigation, and die fuel, respectively. This study provides valuable policy recommendations for promoting synergy between grain production and carbon reduction targets. According to a study Zhang et al., [34], the long-term impact of climatic factors on wheat production in China’s top three provinces (Hebei, Henan, and Shandong) from 1992 to 2020. Using techniques like FMOLS, DOLS, CCR estimators, and Granger causality, it was found that temperature and rainfall negatively impacted wheat production in Henan Province, making it more susceptible to climate change. Conversely, temperature and rainfall positively impacted wheat production in Hebei and Shandong, respectively. Granger causality also revealed significant climatic influences.

### Conceptual framework

**Fig 1:**
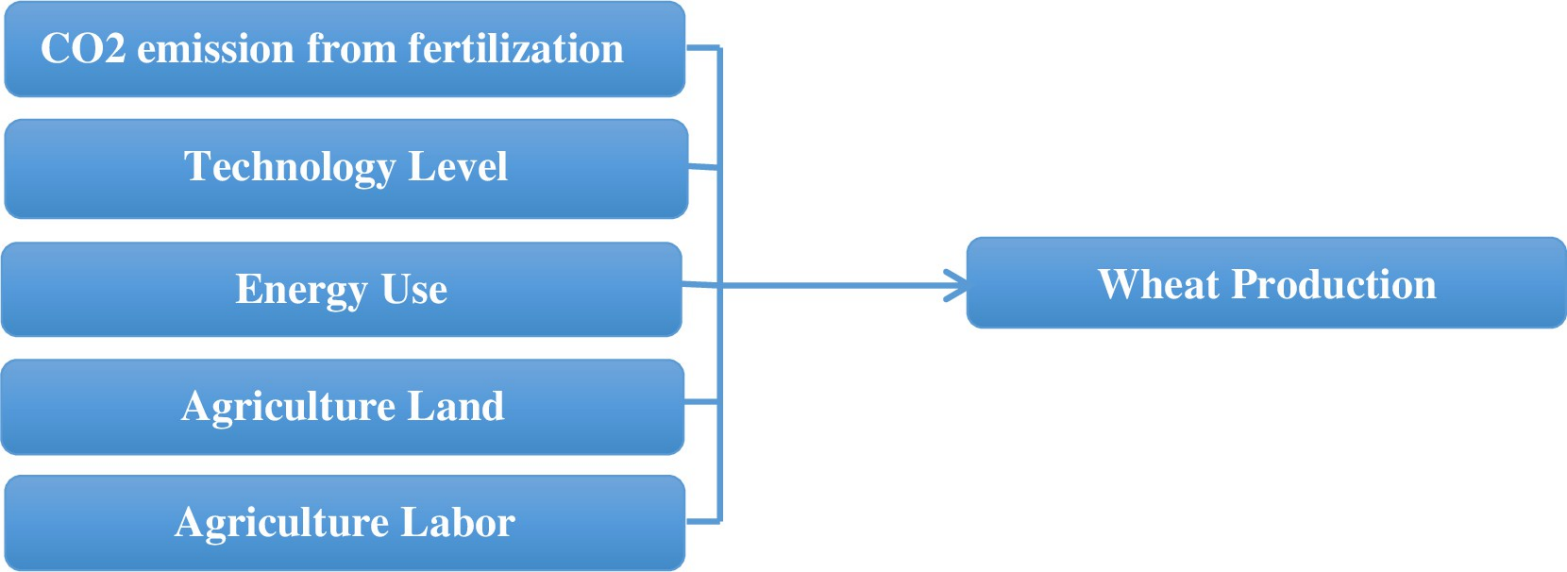
Conceptual framework.

Carbon emissions from fertilizers, agricultural energy consumption, the impact of fertilizers on carbon emissions, and the overall impact of the agricultural system on wheat production are just a few of the problems that need to be researched in depth—the link between carbon emissions from fertilizers and wheat production. To meet expanding wheat demand, many farmers are increasing their use of fertilizers and high-tech agricultural equipment, which raises the need for fossil fuels. This excessive fertilizer usage has two effects on carbon emissions. To begin with, much energy is used to produce fertilizers, which results in smoke-containing carbon emissions. Second, surplus fertilizer combines with water and is lost to the atmosphere due to overwatering. Over-fertilization of wheat fields may increase productivity in the short term. Still, it has long-term consequences such as increased carbon emissions, decreased soil fertility, and more significant production costs. This process’s carbon emissions significantly affect wheat production, as shown in Fig 1.

## Methodology

### Data

The study utilizes time series data from 1990 to 2020 to investigate the impact of factors including CO2 emission from fertilization (CO2EF), technology level (TL), energy use (EU), agriculture land (ALD), and agriculture labor (ALB) on wheat production in Pakistan. The data were collected from an economic survey of Pakistan, the World Bank, and the World Economic Indicators, which provided annual time series data on wheat production in (1000 of tones), (CO2EF) determine the carbon content of fertilizer use default values provided by the IPCC guidelines to estimate the carbon content of the fertilizers. The guidelines provide generic carbon content values for different types of fertilizers. Since there is no specific information on the fertilizer types, we can use the default values as a reasonable approximation. Multiply the total amount of fertilizer used (step 1) by the appropriate carbon content value (step 2) to estimate the carbon emissions. This will give you an annual estimate of carbon emissions from fertilizer use in Pakistan. Technology level, in (total number of tractors used in a year), energy use determined in kg of oil equivalent per capita, agriculture land is selected in% of land area, and agriculture labor is specified in % of male employment.

### Model description

The study conducted experiments in this investigation to determine the long-term and short-term relationships between the variables of interest, shown in Table 1. The study employed an autoregressive distributed lag and Bounds co-integration test to analyze the long-term connection between non-stationary variables. Eliminating error terms requires making all variables in an equation stationary at the same rate. Their non-stationarity does not impede the co-integration of a linear combination of non-stationary variables. Therefore, if a set of variables has a long-run symmetric relationship, it is probable that they are co-integrated [35]. Before estimating ADRL, it is necessary to ensure that the time series data is stationary and that the variables are not co-integrated. Time-series and panel data causality tests that depend on co-integration and integration outcomes can be found in Siddique [36], Rehman [37] and Rizwanullah [38].

**Table 1:** Measurement of study table.

Variable	Code	Measurement
Wheat Production	WP	Wheat Yield in (1000) Metric Tones
CO2 emission from fertilization	CO2EF	kilograms of carbon emitted per unit of nitrogen or phosphorus applied
Technology Level	TL	Number of tractors used in a year
Energy Use	EU	kg of oil equivalent per capita
Agriculture Land	ALD	(% of land area)
Agriculture Labor	ALB	% of male employment

Following is the estimation equation for the ARDL model shown in Eqs 1, 2.


$$y_{t=C_{0}+}\sum_{k=1}^{p}\beta_{k}y_{t-k}+\sum_{j=0}^{t}a_{j+1}x_{t-j}+u_{t}$$ 1

$${\Delta Ln(WP}_{t)=}\beta_{0}+\beta_{1}L_{n}({CO2EF}_{t-1})+\beta_{2}Ln({TL}_{t-1})+\beta_{3}Ln({EU}_{t-i})+\beta_{4}Ln({ALD}_{t-1})+\beta_{5}LnLn({ALB}_{t-1})+{\beta_{1}\sum_{m=1}^{v}\alpha_{i}\Delta Ln({CO2EF}_{t-i})+\beta_{2}\sum_{m=1}^{v}\alpha_{i}\Delta Ln({TL}_{t-i})+\beta }_{3}\sum_{m=1}^{v}\alpha_{i}\Delta Ln({EU}_{t-i})+\sum_{n=1}^{y}\beta_{4}\Delta Ln({ALD}_{t-i})+\sum_{0=1}^{w}\beta_{5}\Delta Ln({ALB}_{t-i})++u_{t}$$ 2

## Results and discussion

### Descriptive statistics

Table 2 displays the variables’ descriptive statistics. Average data for wheat production, CO2 emission from fertilization, technology level, energy use, agricultural land, and agricultural labor in Pakistan are shown in the table below. The minimum and highest values for each variable indicate that WP reaches its maximum value of 4.316, 4.439, 5.174, 2.624, 46.980, and 36.450, respectively. LN_WP has the highest maximum value of 4.426 and the lowest maximum value of 4.163. LN_EU and ALB are negatively skewed, while another variable is positively skewed, as seen by the symmetrical normal distribution of the variables. The Jarque Bera probability value indicates that all variables are consistently distributed.

**Table 2:** Descriptive statistics.

	LN_WP	CO2EF	Ln_TL	LN_EU	ALD	ALB
Mean	4.316	4.439	5.174	2.624	46.980	36.450
Median	4.326	4.344	5.431	2.633	46.942	37.517
Maximum	4.426	5.347	5.656	2.664	48.001	42.823
Minimum	4.163	3.930	4.538	2.567	45.717	28.681
Std. Dev.	0.080	0.364	0.422	0.022	0.728	4.059
Skewness	0.335	1.150	0.298	-0.616	0.289	-0.371
Kurtosis	1.787	3.746	1.267	2.947	1.994	2.085
Jarque-Bera	2.399	7.309	4.200	1.900	1.684	1.734
Probability	0.301	0.026	0.122	0.387	0.431	0.420
Sum	129.482	133.181	155.209	78.731	1409.413	1093.493
Sum Sq. Dev.	0.185	3.841	5.171	0.014	15.353	477.833
Observations	30	30	30	30	30	30

### Normality

**Fig 2:**
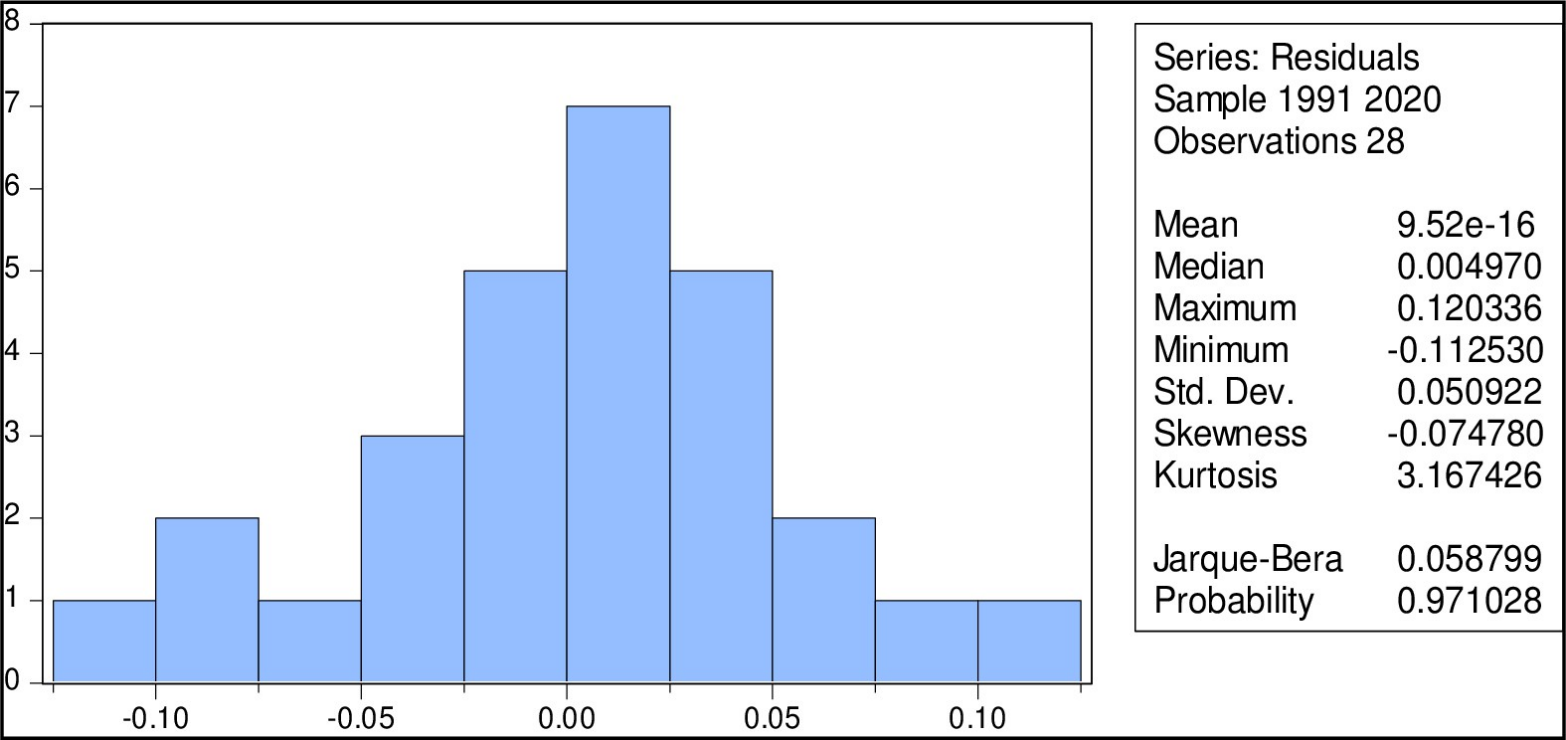
Normality test.

The Jarque-Bera test statistic consistently yields a non-negative result, but a significant deviation from zero indicates that the sample data does not serve a normal distribution. This statistic’s p-value is 0.058799, with a value of 0.971028. The null hypothesis that the data is typically distributed in the scenario described in Fig 2 cannot be rejected.

#### Unit root test

*Augmented Dickey-Fuller*. There are several methods for detecting if an object is at rest. As a result, we conducted the study utilizing standard unit root tests such as the Augmented Dickey-Fuller (ADF). Table 3 shows the results of our tests for stability in variables, including the level and initial differences tests for unit roots, in addition to the constant-only and constant-plus-trend requirements. Using an Augmented Dickey-Fuller (ADF) unit root test, we discovered that all three variables were stationary. Only one variable is significant at the 5% level at a constant. Therefore, at constant and trend wheat production, Co2 emissions from fertilization and agriculture labor are significant at 1% and 5%. However, all variables are significant at 1% and 5% at the first difference. I (1) demonstrate that the variables are not stationary by integrating them into the first order. As a result, conducting a co-integration test as part of an ARDL-bound analysis is critical to look for a long-term link between the variables. Because the variable has a unit root, H0 is false.

**Table 3:** Augmented Dickey-Fuller.

Variables	Level				1^st^ Difference			
	C	Sig. Level	C & T	Sig. Level	C	Sig. Level	C & T	Sig. Level
LN_WP	0.374	no	0.014	**	0.000	***	0.000	***
CO2EF	0.023	**	0.002	***	0.000	***	0.002	***
LN_TL	0.768	no	0.405	No	0.000	***	0.000	***
LN_EU	0.173	no	0.950	no	0.032	**	0.026	***
ALD	0.007	***	0.019	**	0.440	no	0.034	**
ALB	0.999	no	0.120	no	0.000	***	0.010	**

“Notes: (*) Significant at the 10%; (**) Significant at the 5%; (***) Significant at the 1%. and (no) Not Significant

*MacKinnon (1996) one-sided p-values.”

Null Hypothesis: The variable has a unit root.

*Correlation analysis*. The relationships between the variables are shown in Table 4. CO2 emission from fertilization (Co2EF) has a 0.5624 correlation with Wheat Production (MPZ). This is the positive correlation in the matrix. The Technology Level (TL) has a 0.7428 correlation with Wheat Production (MPZ). The correlation between Energy used (EU) and Wheat Production (WP) is 0.8026, which is highly positive. The correlation coefficient between Agriculture Land (ALD) and Wheat Production (WP) is 0.2223. There is a positive correlation between Agriculture Labor (ALB) and Wheat Production (WP), with a correlation coefficient of -0.8887. This is a highly negative correlation.

**Table 4:** Correlation matrix.

Correlation	Ln_WP	CO2EF	Ln_TRC	Ln_EU	ALD	ALB
LN_WP	1.000					
CO2EF	0.562	1.000				
LN_TL	0.743	0.347	1.000			
LN_EU	0.803	0.550	-0.401	1.000		
ALD	0.222	-0.376	0.047	0.078	1.000	
ALB	-0.889	0.542	0.823	-0.625	-0.292	1.000

#### Heteroskedasticity

The probabilities for the Breusch-Pagan and Cook-Weisberg tests for Heteroskedasticity are 0.4417, and the chi-square is 0.3977. At probabilities of 1%, 5%, and 10%, the null hypothesis should be accepted [11, 26]. Consequently, this suggested no issue of Heteroskedasticity in this model.

**Heteroskedasticity Test: Breusch-Pagan-Godfrey**
Ho: Constant variance
Variables: fitted values of NPN
chi2(1) = 0.3977
Prob > chi2 = 0.4417

#### Autocorrelation test

The Durbin-Watson d-statistic is one method to determine autocorrelation, although there are others. The Durbin-Watson d-statistic test determines the levels of autocorrelation. The results indicate that the simulated data are auto-correlated.

Durbin-Watson stat 2.421

#### Co-integration test

*Bounds test approach*. To examine the long-term impact of CO2 emission from fertilization, Technology level, Energy Use, Agriculture Land, and Agriculture Labor on Wheat Production in Pakistan, a co-integration test employing a bound test method at 2 lag has been used. The outcomes are provided in Table 5.

**Table 5:** Bound-test approach.

Test Statistic	Value	k
F-statistic	6.683	5
Critical Value Bounds		
Significance	Lower Bound	Upper Bound
10%,	2.327	3.424
5%,	2.786	3.838
2.50%,	2.968	4.212
1%,	3.584	4.347

Table 5 displays the results of the ARDL bound test for co-integration. The estimated F statistic of 6.68278 (F statistic is greater than the Upper Bound Value) rejects the null hypothesis. It shows that there is a long-term relationship between the variables and demonstrates no co-integration among the variables.

#### The result of the ARDL model

*Short-run impact of variables on wheat production*. The study presents a short-run dynamic and short-run co-integration model within the ARDL Framework, illustrating the convergence rate to equilibrium after equation disruption. The outcomes are summarized in Table 6. The results indicate that the lag CO2 emission from fertilization (Lag_CO2EF) significantly positively short-run impacts wheat production (p<0.05), with a coefficient of 0.163, a t-statistic of 2.785, and a 5% significance level. These findings are consistent with Crost et al., [39]. The log of technology level has a significant positive short-term effect on wheat production nations (p<0.01), with a coefficient of 0.195, a t-statistic of 3.349, and a 1% significance level. These results are consistent with previous findings Aragón et al., [40]. The energy used has a statistically significant positive short-run impact on wheat production countries (p<0.01), with a coefficient of 0.213, a t-statistic of 3.279, and a 1% significance level. These results align with previous findings Carr et al., [41]. Agriculture land has a significant positive short-run influence on wheat production nations (p < 0.01), with a coefficient of 0.072, a t-statistic of 3.039, and a 1% significance level. Results are per earlier research Agnolucci et al., [42]. Agriculture labor has significantly positive short-run effects on wheat production nations (p<0.05), with a coefficient of 0.030, a t-statistic of 2.652, and a 5% significance level. These results align with previous findings Filho et al., [43].

**Table 6:** Short-run impact of variables on wheat production.

Variable	Coefficient	Std. Error	t-Statistic	Prob.	Sig. Level
CO2 emission from fertilization	0.163	0.058	2.785	0.039	**
Technology Level	0.195	0.058	3.349	0.020	****
Energy Used	0.213	0.065	3.279	0.022	***
Agriculture Land	0.072	0.024	3.039	0.029	***
Agriculture Labor	0.030	0.011	2.652	0.045	**
C	0.983	0.198	4.963	0.004	

**“**Notes: (*) Significant at the 10%; (**) Significant at the 5%; (***) Significant at the 1%. And (no) Not Significant.”

*The long-run impact of variables on wheat production*. Table 7 demonstrates that the long-run ARDL results, Lag CO2 emission from fertilization (CO2EF), significantly positive long-run effect wheat production (p<0.05), with a coefficient of 0.333, a t-statistic of 2.158, and a 5% significance level. Results are from earlier research Zhang et al., [44]. Technology level significantly positive long-run affects wheat production (p<0.01), with a coefficient of 0.143, a t-statistic of 3.238, and a 1% significance level. These results are consistent with prior research Hasegawa et al., [45]. Energy use significantly positive long-run impacts on wheat production (p<0.01), with a coefficient of 0.174, a t-statistic of 3.411, and a 1% significance level. These results align with previous findings Wang et al., [46]. Agriculture land has significantly positive long-run effects on wheat production (p<0.05), with a coefficient of 0.051, a t-statistic of 2.684, and a 5% significance level. These results align with previous findings Wang et al., [46]. Agriculture labor has a significantly positive and long-run effect on wheat production (p<0.05), with a coefficient of 0.013, a t-statistic of 2.273, and a 5% significance level. These findings align with the results of Petrović et al., [47].

**Table 7:** Long-run impact of variables on wheat production.

Variable	Coefficient	Std. Error	t-Statistic	Prob.	Sig. Level
Fertilizer Carbon Emission	0.333	0.154	2.158	0.083	*
Technology Level	0.143	0.044	3.238	0.023	***
Energy Used	0.174	0.051	3.411	0.004	***
Agriculture Land	0.051	0.019	2.682	0.044	**
Agriculture Labor	0.013	0.006	2.273	0.039	**

**“Notes:** (*) Significant at the 10%; (**) Significant at the 5%; (***) Significant at the 1%. And (no) Not Significant.

#### Stability test

*Chow breakpoint-test*. The research rejects the null hypothesis, indicating structural change, as indicated by the p-value of 0.0003, which is less than the 1% significance threshold. Tables 7 and 8 findings reveal variations in the slope and coefficient of the two periods.

- Null: There is no structural Change.
- Alternative: There is Structural Change

**Table 8:** Chow breakpoint test: 2010.

Equation Sample: 1991 2020			
F-statistic	9.229	Prob. F(7,17)	0.000
Log likelihood ratio	44.639	Prob. Chi-Square(7)	0.000
Wald Statistic	53.751	Prob. Chi-Square(7)	0.000

## Discussion

This study investigates the impact of multiple factors (carbon dioxide levels from fertilization, energy usage, technology levels, agricultural land, and labor) on wheat production in Pakistan, with significant implications for the nation’s wheat farming industry. The findings indicate that carbon dioxide emissions from fertilization have both short-term and long-term effects on wheat production, echoing previous research. While fertilizers have increased wheat productivity, it is crucial to manage associated CO2 emissions to ensure environmental sustainability. Addressing these challenges requires a blend of sustainable and climate-resilient farming practices, research, and innovation. Similarly, technology levels exhibit significant short-term and long-term impacts on wheat production, as supported by previous research. Technological advancements facilitate higher yields, efficient resource management, and environmentally responsible farming. Realizing these benefits necessitates collaboration between governments, academics, and farmers to promote the widespread adoption of cost-effective agricultural technologies.

Energy usage is another vital factor affecting wheat production, with short-term and long-term impacts aligning with prior studies. Balancing mechanization and water conservation is essential in Pakistan’s wheat production. Policymakers, farmers, and stakeholders should explore options such as renewable energy sources, energy-efficient practices, and legislation promoting sustainable energy use in agriculture. As confirmed by earlier research, agricultural land availability and quality significantly influence wheat production. Sustainable land management, investments in irrigation infrastructure and proper regulations are critical for enhancing Pakistan’s food security and wheat yields. Agricultural labor availability has a short-term impact on wheat production, in line with prior research. As wheat production relies on agricultural labor, policymakers and stakeholders must consider workers’ well-being, skill development, and working conditions to ensure sustainable and productive wheat farming practices amid evolving labor dynamics and mechanization.

## Conclusion

Modern agricultural techniques increase carbon emissions due to their overuse of fertilizer and electricity. The wheat industry’s long-term viability is threatened due to this. Further complicating matters for farmers are falling soil fertility, tainted groundwater supplies, and rising production expenses. More energy is used, and more carbon dioxide is released when farming is done using fossil fuels, chemicals, and high-tech equipment. This adds to the problem of global warming, harms human health, and compounds the difficulties of farming. Due to these consequences, resilient agriculture methods must be implemented immediately. Mitigation of carbon emissions is essential throughout the transition from traditional to modern agriculture. Reduce environmental damage by optimizing fertilizer use, instituting effective energy management methods, and investigating other potential energy sources. Educating farmers on sustainable agricultural practices and appropriate energy sources and raising their awareness of these issues is also essential. Food security and environmental sustainability may be achieved by ecologically friendly methods in the agricultural industry, such as precision farming and using renewable energy.

In conclusion, addressing the impact of carbon emissions on Pakistan’s wheat yield has significant consequences. It requires a comprehensive approach that covers issues such as late fertilizer, energy use, fertilizer application, and equipment (tractors). Adopting sustainable practices and lowering carbon emissions may increase agricultural productivity, environmental protection, and assistance to farmers and the nation.

### Policy implication

Pakistan must adjust its agricultural policy to address carbon emissions from delayed fertilization on wheat output. The policy should align with sustainable development and climate goals, fund agricultural research and extension services, and encourage farmers to adopt low-carbon or carbon-neutral fertilization techniques. Financial incentives and subsidies can help farmers adopt sustainable farming techniques. Training sessions and educational activities can increase farmers’ understanding of the effects of delayed fertilization on the environment. Regional policies should be tailored to local circumstances, and monitoring systems should be implemented to measure carbon emissions. Global alliances can increase capacity and exchange expertise. Policies should restrict high-carbon fertilization techniques and promote water-efficient technologies. Considering their unique challenges, involving farmers and the community in policy formulation is crucial.

### Future research direction and limitation

The study explores substitute fertilization techniques in Pakistani wheat farming, such as biochar, organic fertilizers, and nutrient management strategies. It also explores the development of climate-adaptable wheat cultivars, integrated nutrient and water management strategies, and the social and economic impacts of adopting low-carbon fertilizing methods in Pakistan and different geographical areas worldwide. Acknowledge and deal with Pakistan’s regional variations in soil composition, climate, and farming methods. Thought must be given to regional variation, as research findings and policy recommendations may not be appropriate everywhere. The precision and dependability of study conclusions may be hampered by Pakistan’s dearth of complete and current data on carbon emissions from delayed fertilization. It is necessary to enhance the systems for gathering and exchanging data.

## Supporting information

S1 Data (ZIP)

## References

[1] Apergis N. (2018). Electricity and carbon prices: Asymmetric pass-through evidence from New Zealand. *Energy Sources*, *Part B*: *Economics*, *Planning*, *and Policy*, 13(4), 251–255. 10.1080/15567249.2014.1004002

[2] Nasrullah M., Rizwanullah M., Yu X., Jo H., Sohail M.T. and Liang L. (2021), “Autoregressive distributed lag (ARDL) approach to study the impact of climate change and other factors on rice production in South Korea”, Journal of Water and Climate Change, Vol. 12, 10.2166/wcc.2021.030.

[3] Ahsan F., Chandio A. A., & Fang W. (2020). Climate change impacts on cereal crop production in Pakistan. International Journal of Climate Change Strategies and Management, 12(2), 257–269. 10.1108/IJCCSM-04-2019-0020

[4] Nasrullah M., Liang L., Rizwanullah M., Yu X., Majrashi A., Alharby H. F., et al. (2022) Estimating Nitrogen Use Efficiency,Profitability, and Greenhouse GasEmission Using Different Methods of Fertilization.Front. Plant Sci. 13:869873. 10.3389/fpls.2022.869873. PMC9283998 35845686

[5] Zulfiqar F., Shang J., Nasrullah M. et al. Allocative efficiency analysis of wheat and cotton in district Khanewal, Punjab, Pakistan. GeoJournal (2020). 10.1007/s10708-020-10228-x.

[6] Zulfiqar F., Shang J., Yasmeen S., Wattoo M. U., Nasrullah M., & Alam Q. (2020). Urban agriculture can transform sustainable food security for urban dwellers in Pakistan. GeoJournal. 10.1007/s10708-020-10208-1.

[7] Ndoricimpa A. (2017). Analysis of asymmetries in the nexus among energy use, pollution emissions and real output in South Africa. Energy, 125, 543–551. https://doi.org/https://doi.org/10.1016/j.energy.2017.02.065

[8] Nasrullah M., Liang L. & Rizwanullah M. (2023) Estimating the efficiency gap of maize yield across different irrigation methods in Pakistan. Irrigation and Drainage, 72(1), 166–181. 10.1002/ird.2750

[9] Syed Areeja, Raza Taqi, Talha Tufail Bhatti Neal S. Eash, (2022) Climate Impacts on the agricultural sector of Pakistan: Risks and solutions, Environmental Challenges, 6 (100433), 2667–0100, 10.1016/j.envc.2021.100433.

[10] Seker F., Ertugrul H. M., & Cetin M. (2015). The impact of foreign direct investment on environmental quality: A bounds testing and causality analysis for Turkey. Renewable and Sustainable Energy Reviews, 52, 347–356. https://doi.org/https://doi.org/10.1016/j.rser.2015.07.118

[11] Begum R. A., Sohag K., Abdullah S. M. S., & Jaafar M. (2015). CO2 emissions, energy consumption, economic and population growth in Malaysia. Renewable and Sustainable Energy Reviews, 41, 594–601. https://doi.org/https://doi.org/10.1016/j.rser.2014.07.205

[12] Khoshnevisan B., Shariati H. M., Rafiee S., & Mousazadeh H. (2014). Comparison of energy consumption and GHG emissions of open field and greenhouse strawberry production. Renewable and Sustainable Energy Reviews, 29, 316–324. https://doi.org/https://doi.org/10.1016/j.rser.2013.08.098

[13] Yilmaz I., Akcaoz H., & Ozkan B. (2005). An analysis of energy use and input costs for cotton production in Turkey. Renewable Energy, 30(2), 145–155. https://doi.org/https://doi.org/10.1016/j.renene.2004.06.001

[14] Khoshnevisan B., Rafiee S., Omid M., & Mousazadeh H. (2013). Reduction of CO2 emission by improving energy use efficiency of greenhouse cucumber production using DEA approach. Energy, 55, 676–682. https://doi.org/https://doi.org/10.1016/j.energy.2013.04.021

[15] Ou X., Zhang X., Chang S., & Guo Q. (2009). Energy consumption and GHG emissions of six biofuel pathways by LCA in (the) People’s Republic of China. Applied Energy, 86, S197–S208. https://doi.org/https://doi.org/10.1016/j.apenergy.2009.04.045

[16] Shahbaz M., Shahzad S. J. H., Ahmad N., & Alam S. (2016). Financial development and environmental quality: The way forward. Energy Policy, 98, 353–364. https://doi.org/https://doi.org/10.1016/j.enpol.2016.09.002

[17] Ozturk I. (2017). The dynamic relationship between agricultural sustainability and food-energy-water poverty in a panel of selected Sub-Saharan African Countries. Energy Policy, 107, 289–299. https://doi.org/https://doi.org/10.1016/j.enpol.2017.04.048

[18] Henneman L. R. F., Rafaj P., Annegarn H. J., & Klausbruckner C. (2016). Assessing emissions levels and costs associated with climate and air pollution policies in South Africa. Energy Policy, 89, 160–170. https://doi.org/https://doi.org/10.1016/j.enpol.2015.11.026

[19] Åström S., Tohka A., Bak J., Lindblad M., & Arnell J. (2013). Potential impact on air pollution from ambitious national CO2 emission abatement strategies in the Nordic countries–environmental links between the UNFCCC and the UNECE–CLRTAP. Energy Policy, 53, 114–124. https://doi.org/https://doi.org/10.1016/j.enpol.2012.10.075

[20] Nasrullah M., Rizwanullah M., Yu X. et al. An asymmetric analysis of the impacts of energy use on carbon dioxide emissions in the G7 countries. Environ Sci Pollut Res 28, 43643–43668 (2021). doi: 10.1007/s11356-021-13799-5 33840018

[21] Omri A. (2013). CO2 emissions, energy consumption and economic growth nexus in MENA countries: Evidence from simultaneous equations models. Energy Economics, 40, 657–664. https://doi.org/https://doi.org/10.1016/j.eneco.2013.09.003

[22] Bakhsh K., Rose S., Ali M. F., Ahmad N., & Shahbaz M. (2017). Economic growth, CO2 emissions, renewable waste and FDI relation in Pakistan: New evidences from 3SLS. Journal of Environmental Management, 196, 627–632. doi: 10.1016/j.jenvman.2017.03.029 28364712

[23] Apergis N., & Payne J. E. (2014). Renewable energy, output, CO2 emissions, and fossil fuel prices in Central America: Evidence from a non-linear panel smooth transition vector error correction model. Energy Economics, 42, 226–232. https://doi.org/https://doi.org/10.1016/j.eneco.2014.01.003

[24] Nabavi-Pelesaraei A., Abdi R., & Rafiee S. (2016). Neural network modeling of energy use and greenhouse gas emissions of watermelon production systems. Journal of the Saudi Society of Agricultural Sciences, 15(1), 38–47. https://doi.org/https://doi.org/10.1016/j.jssas.2014.05.001

[25] Jo H., Nasrullah M., Jiang B., Li X., & Bao J. (2021). A Survey of Broiler Farmers’ Perceptions of Animal Welfare and their Technical Efficiency: A Case Study in Northeast China. Journal of Applied Animal Welfare Science, 1–12. doi: 10.1080/10888705.2021.1912605 33843378

[26] Bölük G., & Mert M. (2015). The renewable energy, growth and environmental Kuznets curve in Turkey: An ARDL approach. Renewable and Sustainable Energy Reviews, 52, 587–595. https://doi.org/https://doi.org/10.1016/j.rser.2015.07.138

[27] Dogan E., & Seker F. (2016). Determinants of CO2 emissions in the European Union: The role of renewable and non-renewable energy. Renewable Energy, 94, 429–439. https://doi.org/https://doi.org/10.1016/j.renene.2016.03.078

[28] Mohammadi A., Rafiee S., Mohtasebi S. S., Mousavi Avval S. H., & Rafiee H. (2011). Energy efficiency improvement and input cost saving in kiwifruit production using Data Envelopment Analysis approach. Renewable Energy, 36(9), 2573–2579. https://doi.org/https://doi.org/10.1016/j.renene.2010.10.036

[29] Asongu S., El Montasser G., & Toumi H. (2016). Testing the relationships between energy consumption, CO2 emissions, and economic growth in 24 African countries: a panel ARDL approach. Environmental Science and Pollution Research, 23(7), 6563–6573. doi: 10.1007/s11356-015-5883-7 26635224

[30] Xu Z., Baloch M. A., Danish, Meng F., Zhang J., & Mahmood Z. (2018). Nexus between financial development and CO2 emissions in Saudi Arabia: analyzing the role of globalization. Environmental Science and Pollution Research, 25(28), 28378–28390. doi: 10.1007/s11356-018-2876-3 30083902

[31] Toumi S., & Toumi H. (2019). Asymmetric causality among renewable energy consumption, CO2 emissions, and economic growth in KSA: evidence from a non-linear ARDL model. Environmental Science and Pollution Research, 26(16), 16145–16156. doi: 10.1007/s11356-019-04955-z 30972668

[32] Wang Y., Guo S. S., & Guo P. (2022). Crop-growth-based spatially-distributed optimization model for irrigation water resource management under uncertainties and future climate change. Journal of Cleaner Production, 345, 131182. https://doi.org/https://doi.org/10.1016/j.jclepro.2022.131182

[33] Wang R., Chen J., Li Z., Bai W., & Deng X. (2023). Factors analysis for the decoupling of grain production and carbon emissions from crop planting in China: A discussion on the regulating effects of planting scale and technological progress. Environmental Impact Assessment Review, 103, 107249. https://doi.org/https://doi.org/10.1016/j.eiar.2023.107249

[34] Zhang H, Tang Y, Chandio AA, Sargani GR, Ankrah Twumasi M. Measuring the Effects of Climate Change on Wheat Production: Evidence from Northern China. Int J Environ Res Public Health. 2022 Sep 28;19(19):12341. doi: 10.3390/ijerph191912341 ; PMCID: PMC9565046. 36231641 PMC9565046

[35] Sargani G. R., Deyi Z., Magsi H., Noonari S., Joyo A., Muhammad S., et al. (2018). An Empirical Study of Attitude Towards Entrepreneurial Intention among Pakistan and China Agricultural Graduates in Agribusiness. The International Journal of Business Management and Technology, 2(October), 21–34. www.theijbmt.com

[36] Siddique H. M. A. (2017). Impact of Financial Development and Energy Consumption on CO 2 Emissions: Evidence from Pakistan Hafiz Muhammad Abubakar Siddique Federal Urdu University Islamabad, Pakistan. Bulletin of Business and Economics, 6(2), 68–73.

[37] Rehman A., Chandio A. A., Hussain I., & Jingdong L. (2019). Fertilizer consumption, water availability and credit distribution: Major factors affecting agricultural productivity in Pakistan. Journal of the Saudi Society of Agricultural Sciences, 18(3), 269–274. https://doi.org/https://doi.org/10.1016/j.jssas.2017.08.002

[38] Rizwanullah M., Nasrullah M. & Liang L. On the asymmetric effects of insurance sector development on environmental quality: challenges and policy options for BRICS economies. Environ Sci Pollut Res 29, 10802–10811 (2022). doi: 10.1007/s11356-021-16364-2 34532796

[39] Crost B., Duquennois C., Felter J. H., & Rees D. I. (2018). Climate change, agricultural production and civil conflict: Evidence from the Philippines. Journal of Environmental Economics and Management, 88, 379–395. 10.1016/j.jeem.2018.01.005

[40] Aragón F. M., Oteiza F., & Rud J. P. (2021). Climate Change and Agriculture: Subsistence Farmers’ Response to Extreme Heat. American Economic Journal: Economic Policy, 13(1). 10.1257/pol.20190316

[41] Carr T. W., Mkuhlani S., Segnon A. C., Ali Z., Zougmoré R., Dangour A. D., et al. (2022). Climate change impacts and adaptation strategies for crops in West Africa: A systematic review. Environmental Research Letters, 17(5). 10.1088/1748-9326/ac61c8

[42] Agnolucci P., Rapti C., Alexander P., De Lipsis V., Holland R. A., Eigenbrod F., et al. (2020). Impacts of rising temperatures and farm management practices on global yields of 18 crops. Nature Food, 1(9), 562–571. doi: 10.1038/s43016-020-00148-x 37128016

[43] Filho W. L., Setti A. F. F., Azeiteiro U. M., Lokupitiya E., Donkor F. K., Etim N. A. N. A., et al. (2022). An overview of the interactions between food production and climate change. Science of the Total Environment, 838(June). doi: 10.1016/j.scitotenv.2022.156438 35660578

[44] Zhang Z., Wei J., Li J., Jia Y., Wang W., Li J., et al. (2022). The impact of climate change on maize production: Empirical findings and implications for sustainable agricultural development. Frontiers in Environmental Science, 10(September), 1–10. 10.3389/fenvs.2022.954940

[45] Hasegawa T., Wakatsuki H., Ju H., Vyas S., Nelson G. C., Farrell A., et al. (2022). A global dataset for the projected impacts of climate change on four major crops. Scientific Data, 9(1), 58. doi: 10.1038/s41597-022-01150-7 35173186 PMC8850443

[46] Wang X., Zhao C., Müller C., Wang C., Ciais P., Janssens I., et al. (2020). Emergent constraint on crop yield response to warmer temperature from field experiments. Nature Sustainability, 3(11), 908–916. 10.1038/s41893-020-0569-7

[47] Petrović G., Karabašević D., Vukotić S., Mirčetić V., & Radosavac A. (2020). The impact of climate change on the corn yield in Serbia. Acta Agriculturae Serbica, 25(50), 133–140. 10.5937/aaser2050133p

